# Supplementary material for: Mutational scanning pinpoints distinct binding sites of key ATGL regulators in lipolysis
Source: Nat Commun. 2024 Mar 21;15:2516. doi: 10.1038/s41467-024-46937-x (PMC10958042; doi:10.1038/s41467-024-46937-x)
Supplement: Supplementary file 1 — Supplementary Information [file 41467_2024_46937_MOESM1_ESM.pdf]

Supplemental Information

Mutational scanning pinpoints distinct binding sites of key ATGL regulators in lipolysis

Johanna M. Kohlmayr, Gernot F. Grabner, Anna Nusser, Anna Höll, Verina Manojlović, Bettina Halwachs, Sarah Masser, Evelyne Jany-Luig, Hanna Engelke, Robert Zimmermann, Ulrich Stelzl

Suppl. Figure 1: Expression of ATGL constructs in HEK293T cells for Co-IP analysis.

Suppl. Figure 2: Protein expression and phenotype of T-REx™-293 cells stably expressing PLIN1 or PLIN5.

Suppl. Figure 3:

Single cell confocal images of stained, live T-REx™-293 cells stably expressing doxycycline induced PLIN1.

Suppl. Figure 4:

Single cell confocal images of stained, live T-REx™-293 cells stably expressing doxycycline induced PLIN5.

Suppl. Figure 5:

Confocal images of stained, live T-REx™-293 cells stably expressing doxycycline induced PLIN1.

Suppl. Figure 6:

Confocal images of stained, live T-REx™-293 cells stably expressing doxycycline induced PLIN5.

Suppl. Figure 1

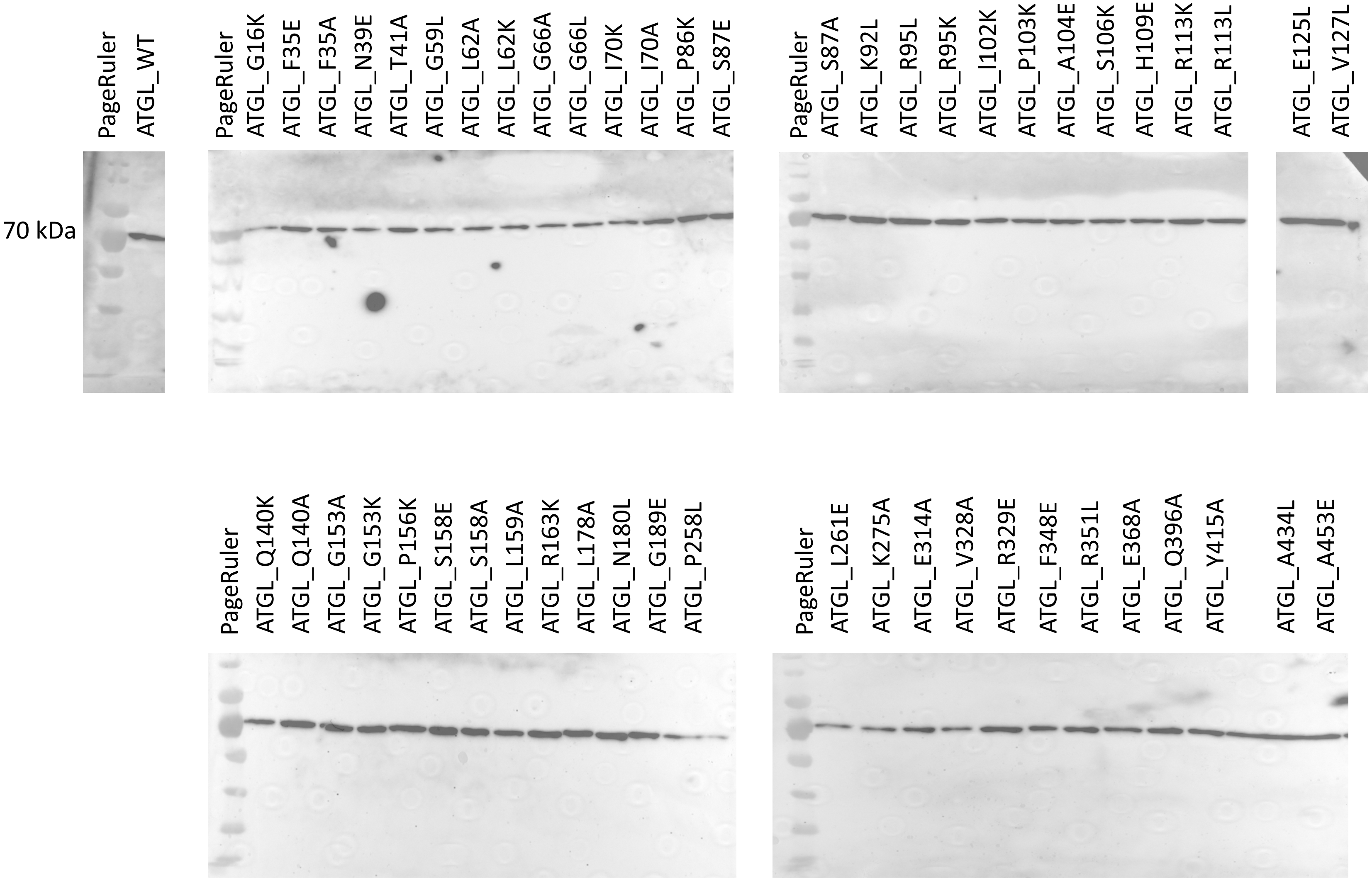

Kohlmayr et al, **Suppl. Figure 1**:
Expression of ATGL constructs in HEK293T cells for Co-IP analysis. Cells were lysed in SDS-free RIPA2 buffer, and cleared samples were loaded on a 10% SDS-gel and blotted on a nitrocellulose membrane. Immunodetection was performed using an Ab against Protein A (1:3000, anti-rabbit-IgG-HRP linked, NA934, GE Healthcare/Amersham).

Suppl. Figure 2

A

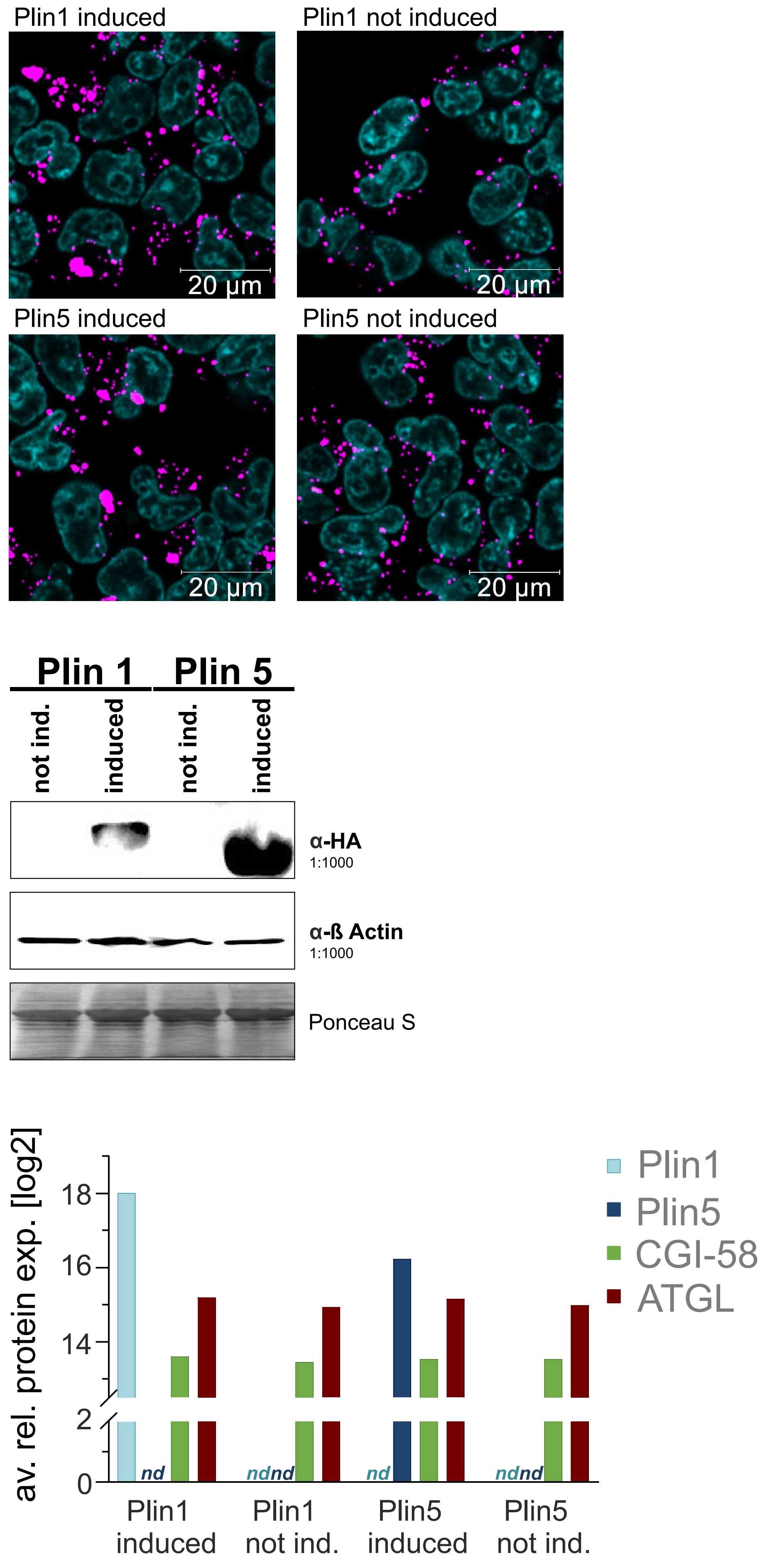

Kohlmayr et al, **Suppl. Figure** 2:
Protein expression and phenotype of T-REx™-293 cells stably expressing PLIN1 or PLIN5.

Top: Confocal images of T-REx™-293 cells stably expressing PLIN1 or PLIN5 (induced) or not induced. Plin1/5 expression was induced with doxycycline (2µg/ml) for 24 hours and then treated with 200µM oleic acid for 6h. Pictures are merged fluorescent images of live cells stained with Hoechst in cyan and Bodipy 558/568 C12 in magenta.

Middle: Western blot of whole cell extracts of T-REx™-293 cells expressing PLIN1 or PLIN5 (induced) or not induced. Cells were treated as in A. Top: PVDF membrane developed with anti-HA.11 Epitope Tag antibody (1:10000, 901501, Biolegend);
Middle PVDF membrane developed with anti-ß Actin (clone AC-15) antibody (1:5000, A5441, Sigma); Bottom: Ponceau stained PVDF membrane.

Bottom: Mass spectrometry based relative protein quantification of PLIN1, PLIN5, CGI-58 and ATGL from total cell extract of T-REx™-293 cells stably expressing doxycycline inducible PLIN1 or PLIN5 (induced and not induced).

Suppl. Figure 3

Kohlmayr et al, **Suppl. Figure** 3:
Single cell confocal images of stained, live T-REx™-293 cells stably expressing doxycycline induced PLIN1.
Cells were transiently transfected with YFP-ATGL variants and PLIN1/5 expression simultaneously induced with doxycycline (2µg/ml). After 24 hours cells were treated with 200µM oleic acid for 6h. Pictures are merged fluorescent images with Hoechst in cyan, YFP-ATGL in yellow and Bodipy 558/568 C12 (D3835, FisherScientific) in magenta, next to the three separated channels. The experiments were repeated three times with similar outcome.

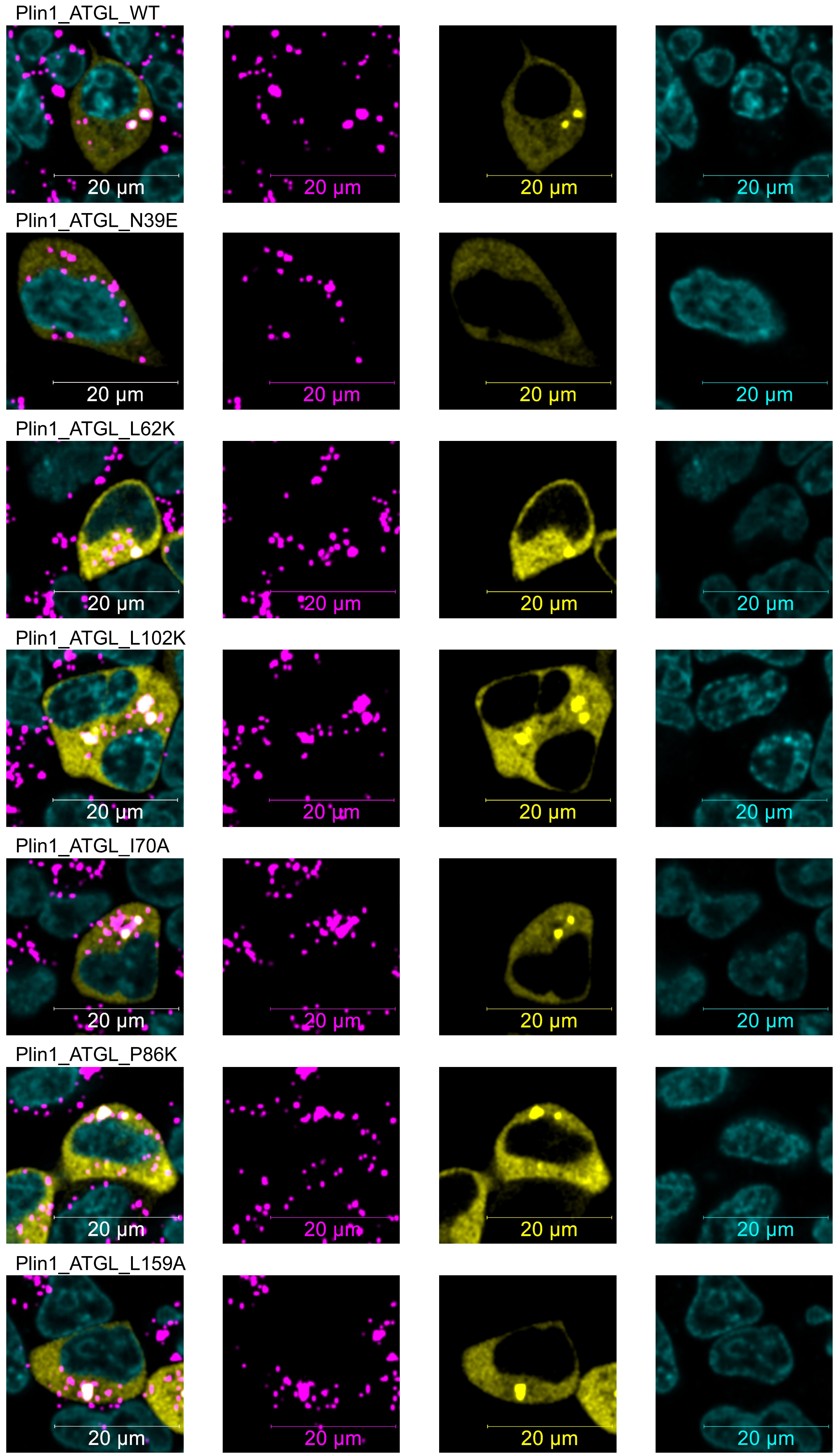

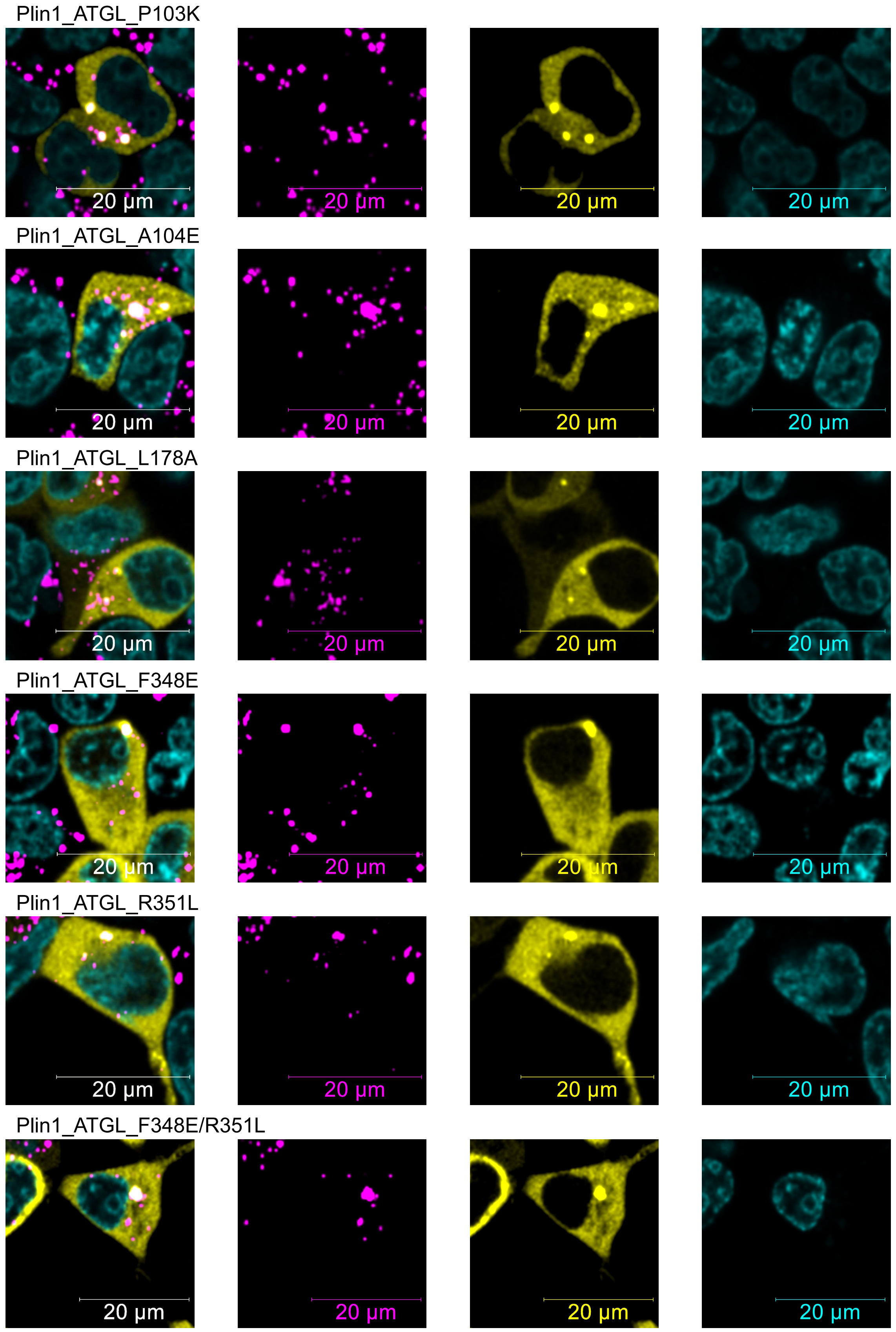

Suppl. Figure 4

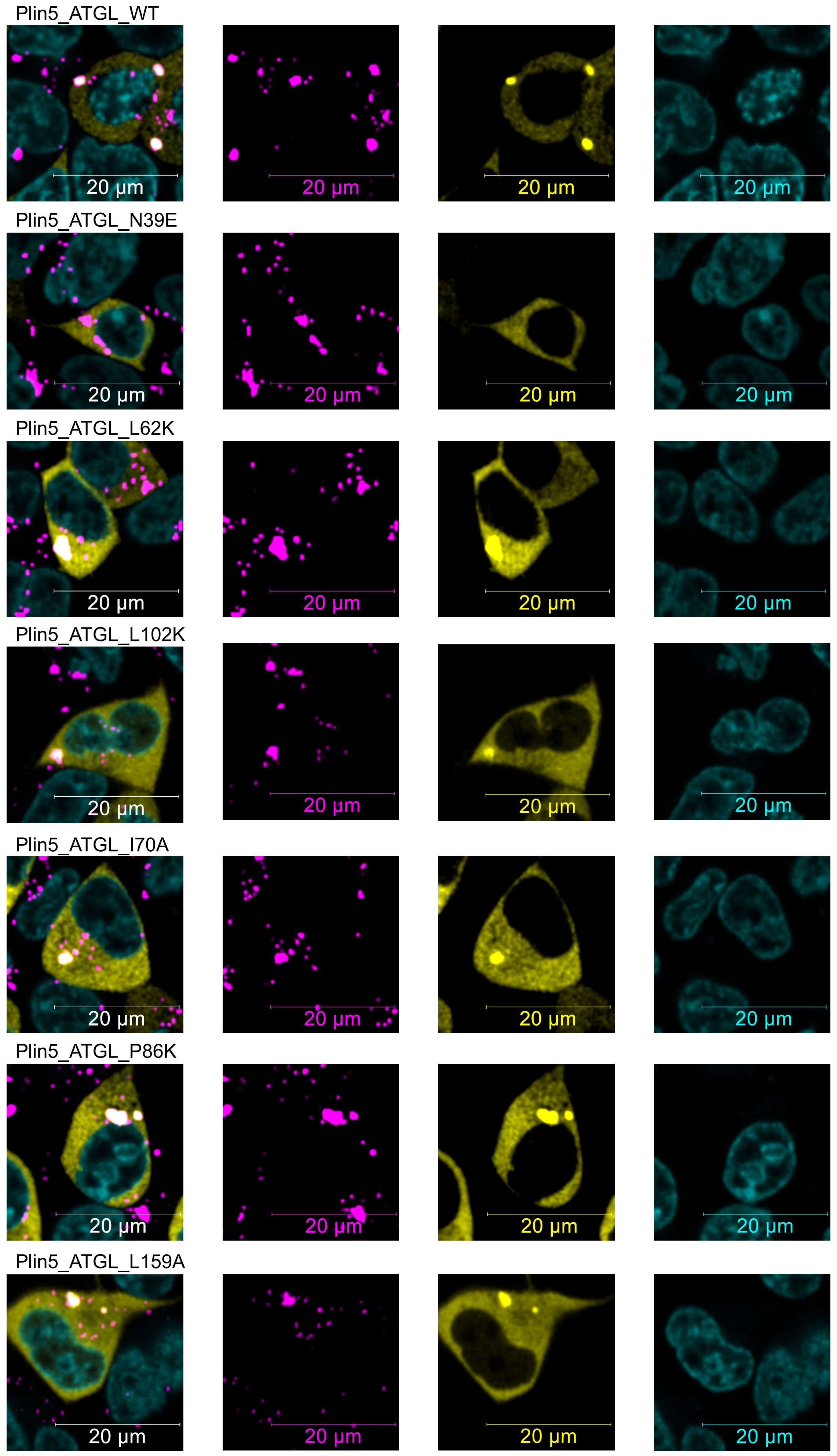

Kohlmayr et al, **Suppl. Figure** 4:
Single cell confocal images of stained, live T-REx™-293 cells stably expressing doxycycline induced PLIN5.
Cells were transiently transfected with YFP-ATGL variants and PLIN1/5 expression simultaneously induced with doxycycline (2µg/ml). After 24 hours cells were treated with 200µM oleic acid for 6h. Pictures are merged fluorescent images with Hoechst in cyan, YFP-ATGL in yellow and Bodipy 558/568 C12 (D3835, FisherScientific) in magenta, next to the three separated channels. The experiments were repeated three times with similar outcome.

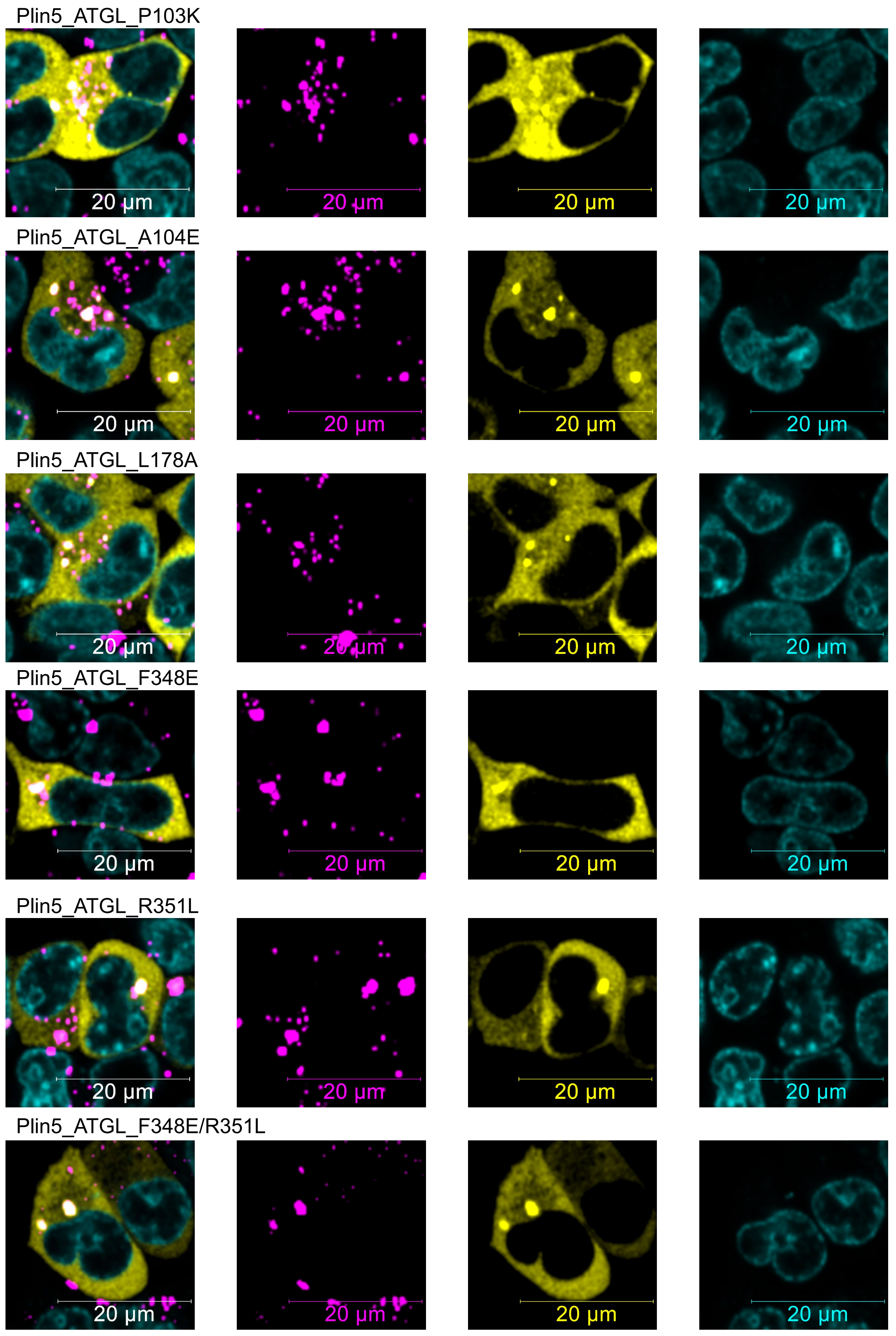

Suppl. Figure 5

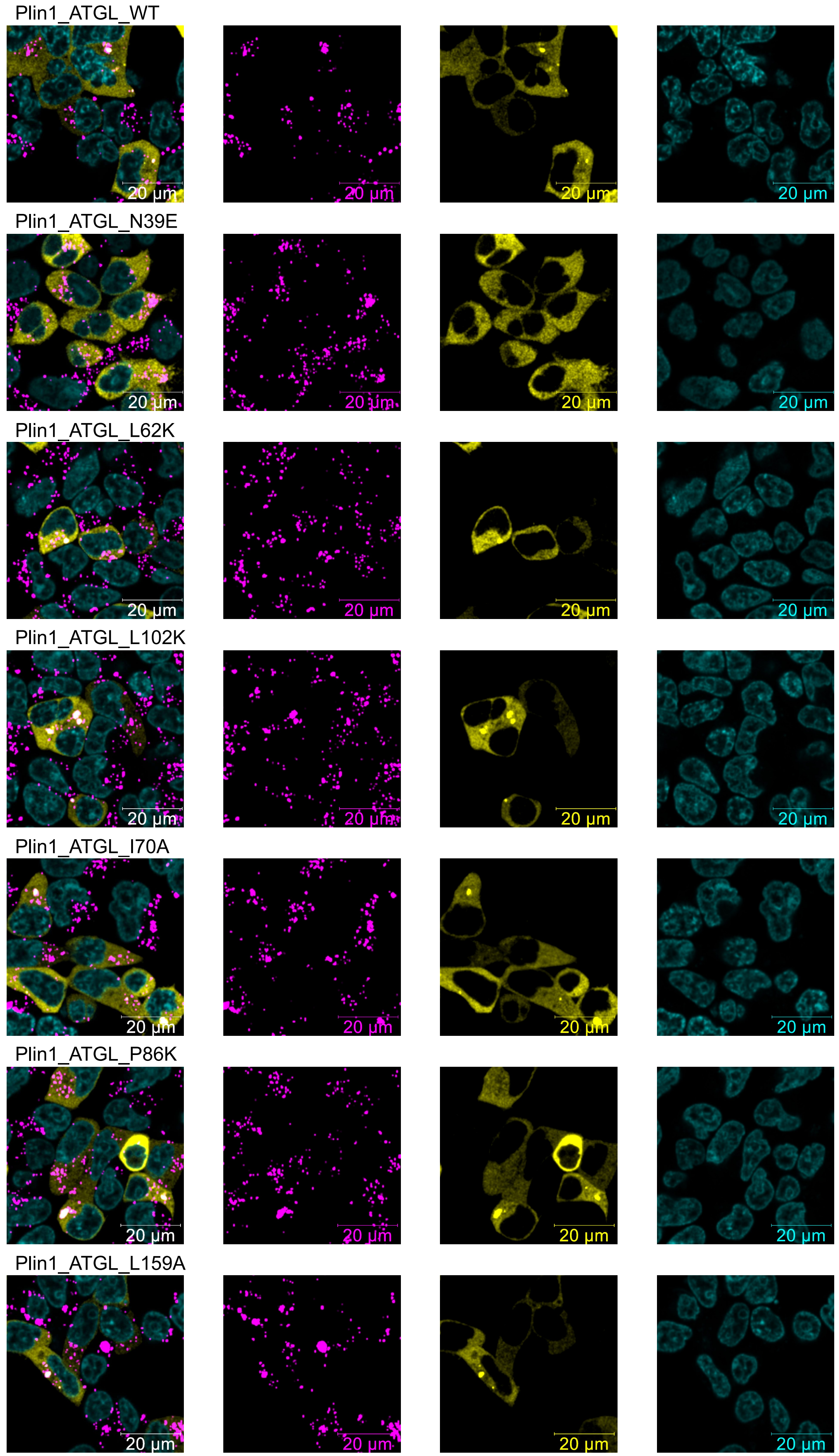

Kohlmayr et al, **Suppl. Figure** 5:
Confocal images of stained, live T-REx™-293 cells stably expressing doxycycline induced PLIN1.
Cells were transiently transfected with YFP-ATGL variants and PLIN1/5 expression simultaneously induced with doxycycline (2µg/ml). After 24 hours cells were treated with 200µM oleic acid for 6h. Pictures are merged fluorescent images with Hoechst in cyan, YFP-ATGL in yellow and Bodipy 558/568 C12 (D3835, FisherScientific) in magenta, next to the three separated channels. The experiments were repeated three times with similar outcome.

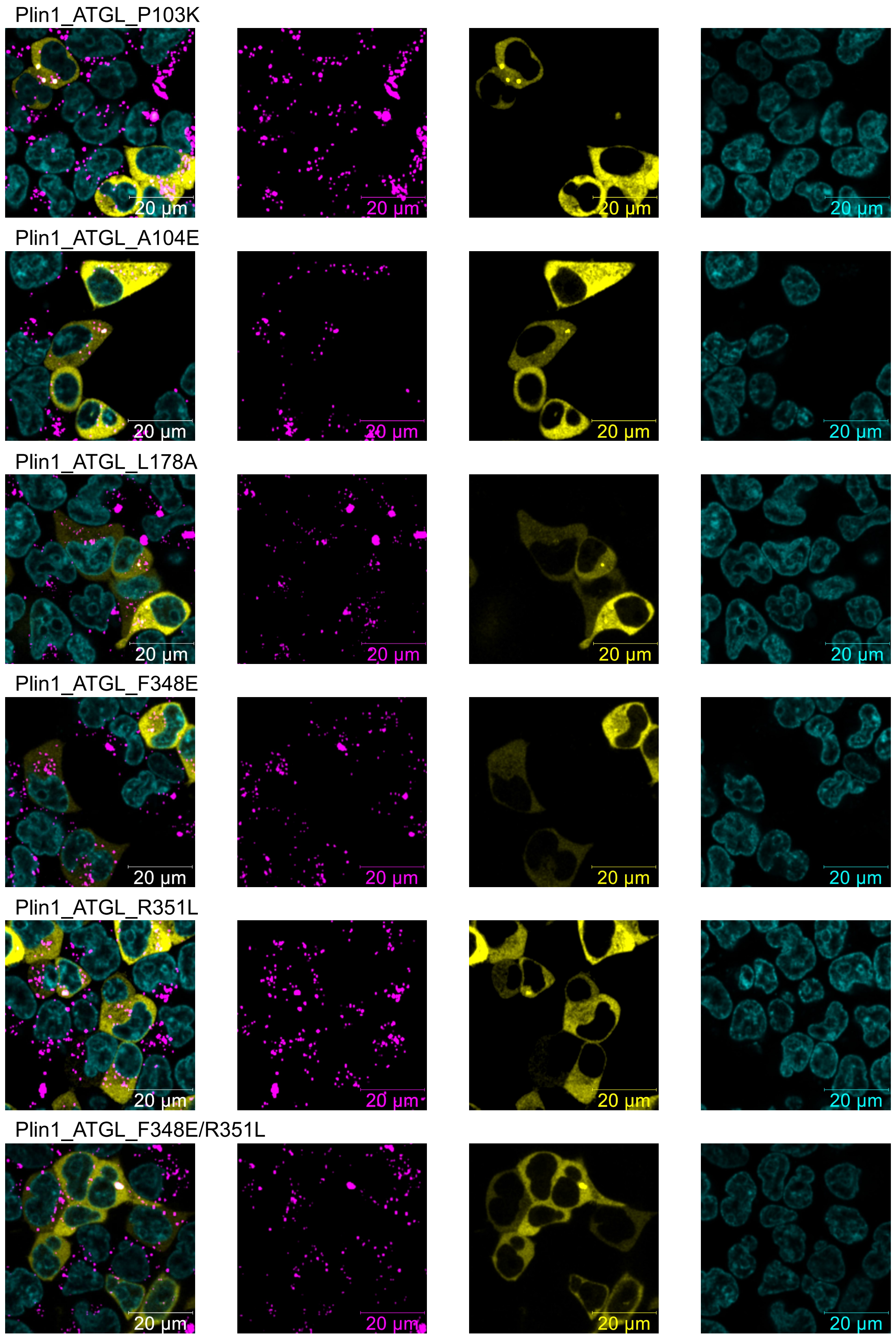

Suppl. Figure 6

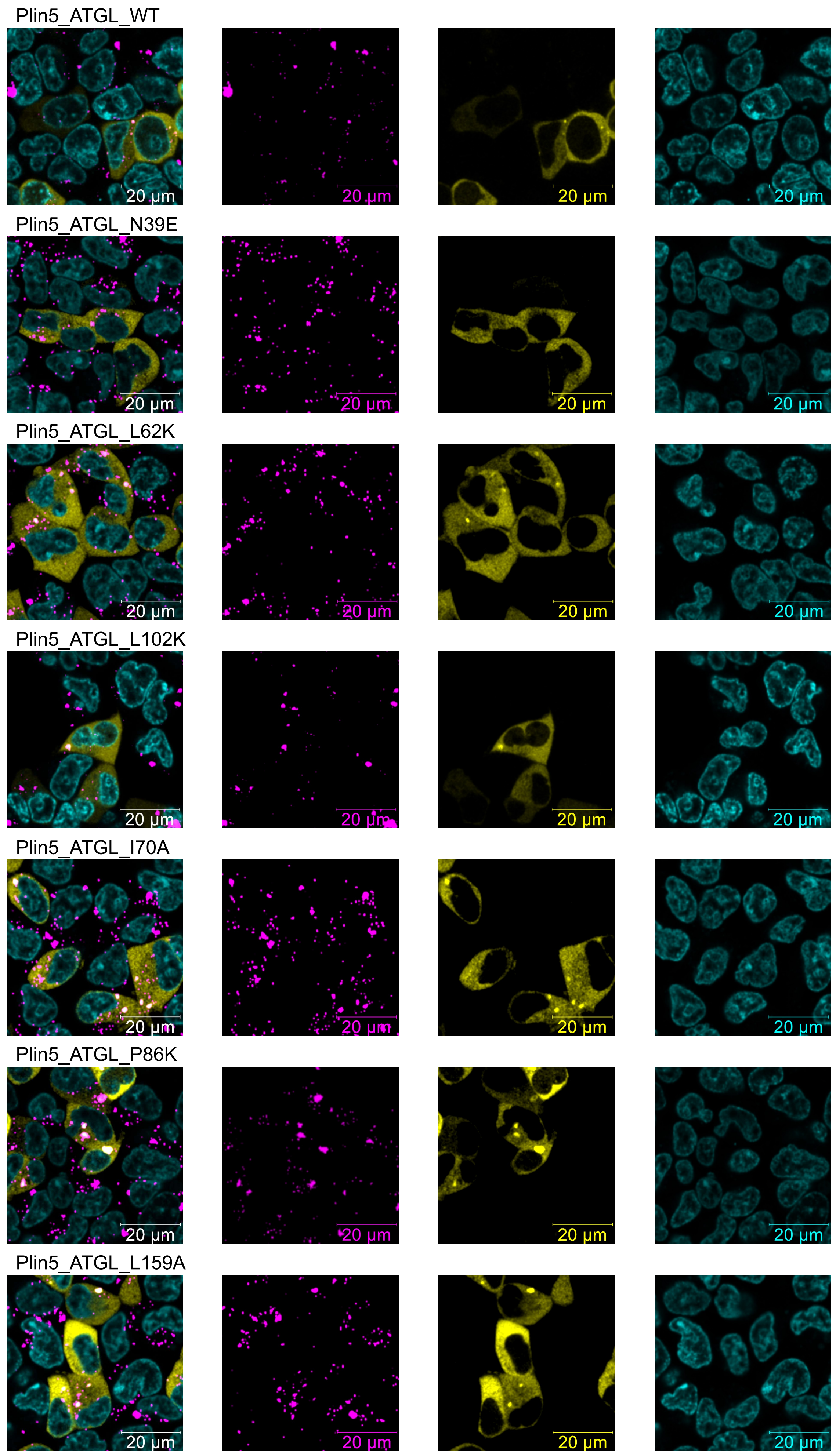

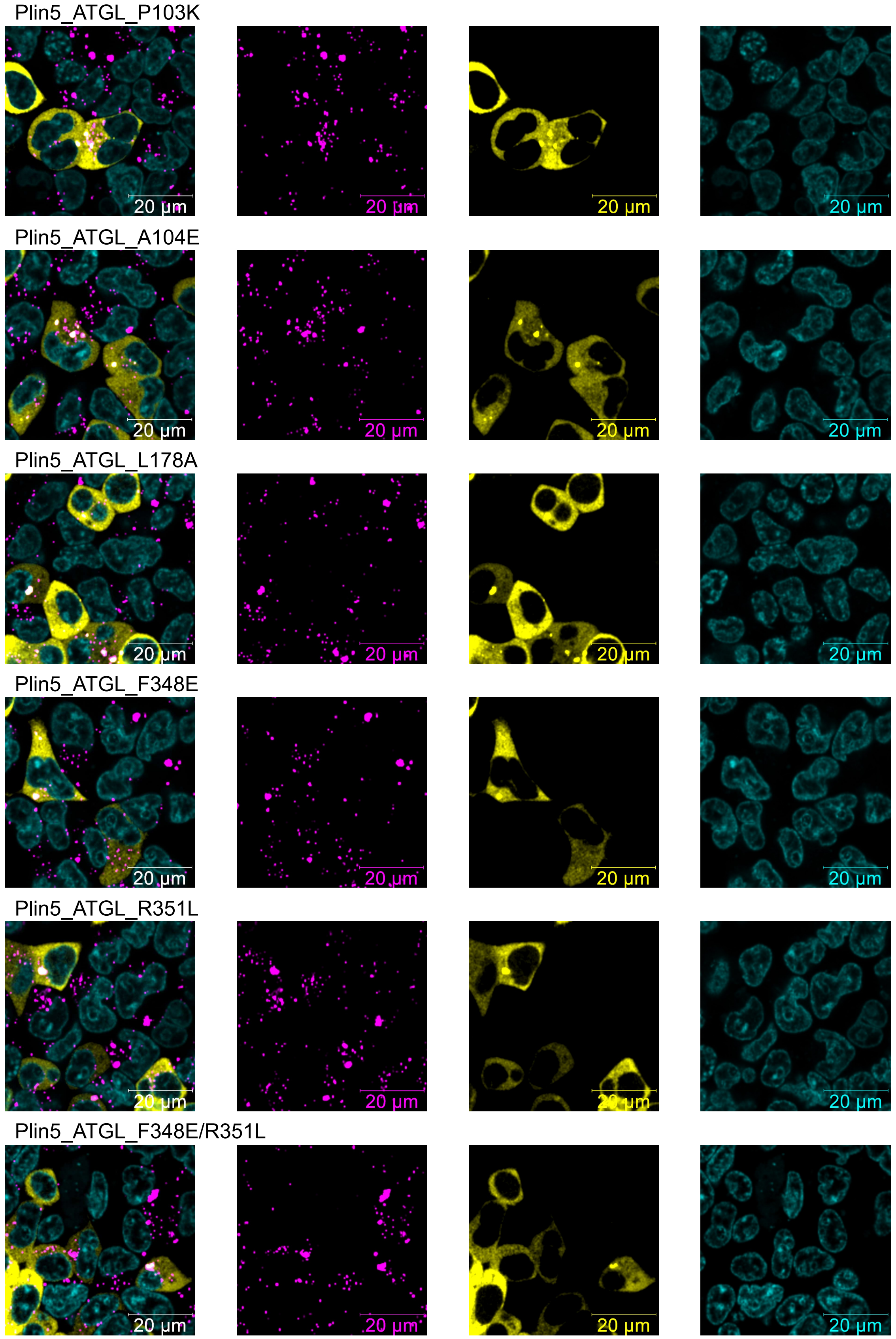

Kohlmayr et al, **Suppl. Figure** 6:
Confocal images of stained, live T-REx™-293 cells stably expressing doxycycline induced PLIN5.
Cells were transiently transfected with YFP-ATGL variants and PLIN1/5 expression simultaneously induced with doxycycline (2µg/ml). After 24 hours cells were treated with 200µM oleic acid for 6h. Pictures are merged fluorescent images with Hoechst in cyan, YFP-ATGL in yellow and Bodipy 558/568 C12 (D3835, FisherScientific) in magenta, next to the three separated channels. The experiments were repeated three times with similar outcome.
