# Supplementary material for: Mutational scanning pinpoints distinct binding sites of key ATGL regulators in lipolysis
Source: Nat Commun. 2024 Mar 21;15:2516. doi: 10.1038/s41467-024-46937-x (PMC10958042; doi:10.1038/s41467-024-46937-x)
Supplement: Supplementary file 3 — Description of Additional Supplementary Files [file 41467_2024_46937_MOESM3_ESM.pdf]

## Description of Additional Supplementary Files:

**Supplementary Data 1:** Protein pairs in interaction perturbation screening

**Supplementary Data 2:** Cutoff values used in sequence data processing

**Supplementary Data 3:** 52 ATGL single amino acid mutants

**Supplementary Data 4:** Co-IP results of the 52 ATGL single amino acid mutants (log2 fold change)

**Supplementary Data 5:** Quantification of cells with LDs
